# Chemotherapy for post‐menopausal women with early breast cancer seems not to result in clinically significant changes in thyroid function

**DOI:** 10.1002/cam4.70015

**Published:** 2024-08-06

**Authors:** Djordje Marina, Kristian Buch‐Larsen, Linn Gillberg, Mads Albrecht Andersen, Michael Andersson, Åse Krogh Rasmussen, Peter Schwarz

**Affiliations:** ^1^ Department of Endocrinology and Metabolism Copenhagen University Hospital, Rigshospitalet Copenhagen Denmark; ^2^ Department of Biomedical Sciences University of Copenhagen Copenhagen Denmark; ^3^ Department of Oncology Centre for Cancer and Organ Diseases, Copenhagen University Hospital, Rigshospitalet Copenhagen Denmark; ^4^ Faculty of Health Sciences University of Copenhagen Copenhagen Denmark

**Keywords:** adjuvant chemotherapy, early breast cancer, postmenopausal women, thyroid function

## Abstract

**Objective:**

Adjuvant chemotherapy is often indicated in patients diagnosed with early breast cancer (EBC). Among others, weight gain is one of the observed side effects of both chemotherapy and other cancer treatments; however, the mechanism is not well‐described. In this study, we aimed to assess thyroid function before and shortly after the course of chemotherapy for EBC.

**Methods:**

This is a prospective cohort study of women diagnosed with EBC. The main outcome was the thyroid function and body weight before and after completing chemotherapy. Secondary outcomes were the presence of thyroid autoantibodies and treatment radiation dosage. We included 72 patients treated with adjuvant chemotherapy, whereas 59 patients also received supraclavicular locoregional radiotherapy. Triple‐negative breast cancer (BC) patients receiving chemoimmunotherapy were excluded.

**Results:**

After the chemotherapy, we observed an increase in thyroid‐stimulating hormone (*p* = 0.03) and a decrease in free‐thyroxine (*p* = 0.0006), with no significant weight change. The prevalence of autoimmune thyroiditis was low. On average 3 months post‐chemo, we found no statistically significant difference in the thyroid function of women treated versus not treated with supraclavicular locoregional radiotherapy.

**Conclusions:**

Although statistically significant changes in thyroid hormones were observed, this study suggests no obvious clinically significant changes in thyroid function in women with early BC after the course of chemotherapy. The decrease in thyroid function was not related to autoimmunity, non‐thyroidal illness, radiotherapy, or high‐dose corticosteroids. Further studies with a longer follow‐up of thyroid function after adjuvant chemotherapy and supraclavicular locoregional radiotherapy are needed.

## Graphical abstract

Our data suggest statistically, but no obvious clinically significant changes in thyroid function in women with early breast cancer after the course of chemotherapy. As far as we are aware, this study is the first with the power to evaluate the thyroid function in this patient group.

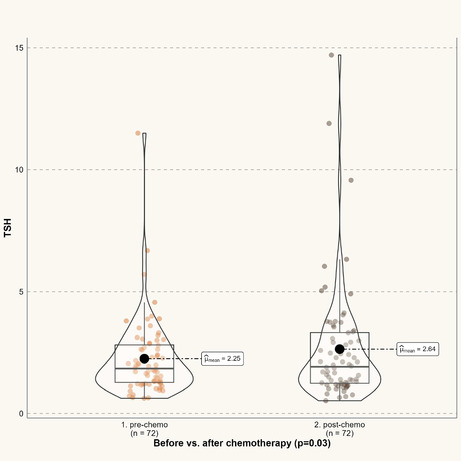

## 1 INTRODUCTION

Breast cancer (BC) is the most common cancer worldwide.^1^ Since 2000, the occurrence of BC in women has gradually increased by 0.5% per year, probably being influenced by advanced screening and diagnostics in the early stage of the disease^2^ but also by excess body weight and a general decrease in the fertility rate.^3^ Due to advancing therapeutic options, the overall survival rate is high, with a steadily declining mortality rate of 43% from 1989.^4^ Depending on tumor staging and BC type (estrogen‐receptor positive and/or human epidermal growth factor receptor‐2 (HER‐2) positive) different therapy options are available. Adjuvant chemotherapy in combination with surgery, targeted drug therapy, localized radiotherapy (RT), and anti‐estrogen therapy is shown to reduce the recurrence and mortality of BC.^5^

Weight gain is a common observation during chemo‐, targeted drug‐ and anti‐estrogen treatment.^6^ Obesity at diagnosis of BC is found to be related to a worse disease‐free^7^ and overall survival of BC,^8^ increased risk of local recurrence,^9^ and the occurrence of other obesity‐related comorbidities.^10^ Chemotherapy has been observed to increase mean weight by 2–5 kg in around 50% of patients, and up to 20% experience even more significant weight gain (10–20 kg).^11^ A higher level of estrogen in obesity is an independent risk factor for BC or a poorer prognosis of already diagnosed patients. Many factors, such as changes in metabolic rate^12^ and physical activity^13^ due to physiological disturbances related to BC diagnosis might be an explanation for the reported weight gain, but they are not yet scientifically confirmed. A meta‐analysis on weight change during chemotherapy in BC included 2620 women from 25 publications.^14^ Despite heterogeneity between studies, this meta‐analysis emphasized therapy‐associated weight gain as a remaining and significant problem.^14^

Both overt and subclinical hypothyroidism are risk factors for weight gain,^15^, ^16^ however, a newer review on the topic is questioning this hypothesis.^17^ It seems that the mechanisms behind weight gain are far more complicated, involving the importance of metabolic active fat tissue, resting metabolic rate, and the direct influence of weight changes on thyroid‐stimulating hormone levels.^17^ A recent systematic review showed alterations in thyroid function during chemotherapy for BC, thus relation to weight was not discussed.^18^ Furthermore, BC‐related RT increases the risk of hypothyroidism. A newly published meta‐analysis reports the highest risk of hypothyroidism after RT in supraclavicular fields compared to RT of the breast and chest wall only.^19^ Most reports on hypothyroidism show the development of hypothyroidism with a median of 8–27 months after RT, but mostly in patients with head‐ and neck cancer.^20^, ^21^, ^22^, ^23^, ^24^ As weight gain and thyroid function (e.g., hypothyroidism) are closely related, possible thyroid dysfunction might be associated with the therapy‐induced weight gain observed in patients with BC. Therefore, we aimed to prospectively evaluate thyroid function in postmenopausal women with early breast cancer (EBC) after adjuvant chemotherapy.

## 2 MATERIALS AND METHODS

### 2.1 Materials

**FIGURE 1:**
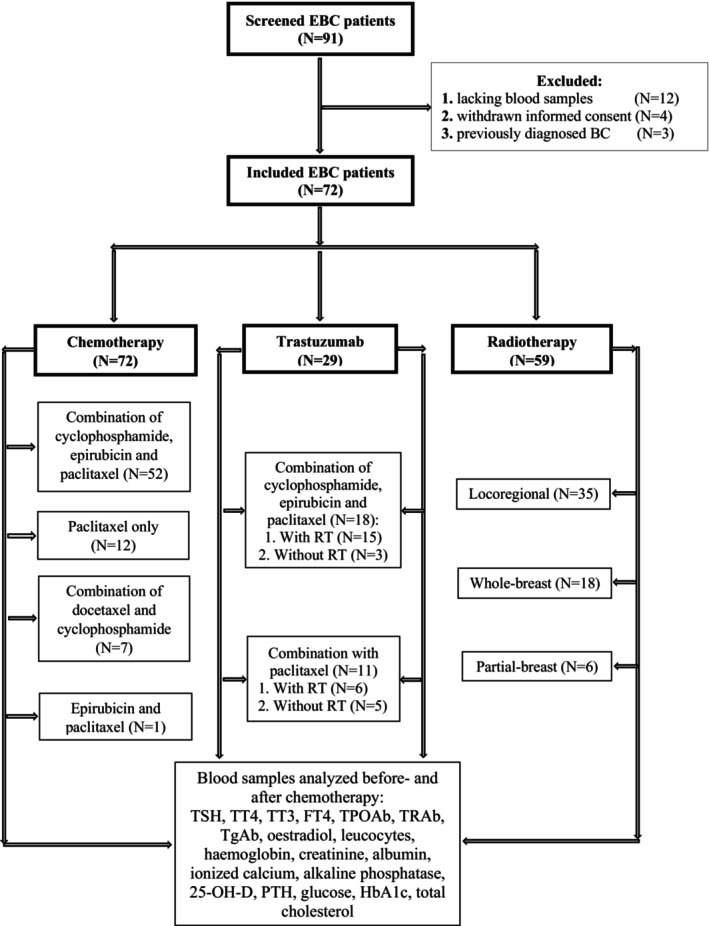
Participant flow diagram. 25‐OH‐D, 25‐hydroxy vitamin D2; BC, breast cancer; EBC, early breast cancer; FT4, free‐thyroxine; HbA1c, glycated hemoglobin; PTH, parathyroid hormone; RT, radiotherapy; TT3, total triiodothyronine; TT4, total thyroxine; TgAb, thyroid‐globulin antibody; TSH, thyroid‐stimulating hormone; TPOAb, thyroid‐peroxidase antibody; TRAb, TSH‐receptor antibody.

Post‐menopausal women diagnosed with EBC (a tumor found only in the breast or nearby lymph nodes) from the Department of Oncology, Rigshospitalet, were consecutively invited to participate in the study and were evaluated before and after the course of chemo‐ and radiation therapy. Inclusion criteria were post‐menopausal women aged 50–70 years, diagnosed with EBC eligible for adjuvant chemotherapy. Exclusion criteria were pre‐menopausal women, preexisting endocrine disease (e.g., thyroid‐ and pituitary disease, diabetes mellitus), disseminated or recurrent BC, triple‐negative BC treated with chemoimmunotherapy, and cancer‐related treatment before the current diagnosis of EBC. We included in total 72 post‐menopausal women with EBC treated with chemotherapy, of whom 29 received a targeted cancer drug trastuzumab, and 59 received RT (flow diagram, Figure 1).

### 2.2 Methods

Fasting blood samples were obtained before chemotherapy from venipuncture between 8:00 and 10:00 AM in the antecubital vein and processed and analyzed at the central laboratory at Rigshospitalet, Denmark. We collected the next blood samples after finalizing chemotherapy and RT. The evaluation of thyroid function included serum (s‐) thyroid‐stimulating hormone (s‐TSH), s‐total thyroxine (s‐TT4), s‐free‐thyroxine (s‐FT4), s‐total triiodothyronine (s‐TT3), s‐thyroid‐globulin antibody (s‐TgAb), s‐thyroid‐peroxidase antibody (s‐TPOAb), and s‐TSH‐receptor antibody (s‐TRAb). Thyroid dysfunction was evaluated based on local reference ranges: overt hypo/hyperthyroidism as low/high TT4 or FT4 associated with an increased/decreased TSH, respectively, whereas subclinical hypothyroidism as normal TT4 or FT4 concentration associated with an inappropriate elevation of TSH. Analyses of TT3 were used to exclude low T3 syndrome/non‐thyroidal illness defined as low TT3/TT4 level in combination with an inappropriately low TSH. Positive autoantibody (TgAb, TPOAb, or TRAb) was suggestive of autoimmune thyroid disease.

The type and performance characteristics of the assays used for the hormone measurements are shown in Table S1. Thyroid parameters (s‐TSH, s‐TT4, s‐FT4, s‐TT3) were analyzed by immunohistochemistry, s‐TgAb, and s‐TPOAb by immunofluorometry, whereas s‐TRAb was analyzed by electrochemiluminescence immunoassay. Further details (reference values, detection limits, intra‐, and total assay imprecision, and equipment) are shown in Table S1.

Further to thyroid evaluation, we measured safety parameters for the evaluation of another endocrine dysfunction influencing metabolism. We assessed blood (b‐) hemoglobin, s‐leucocytes, s‐albumin, s‐creatinine, s‐ionized calcium, s‐parathyroid hormone (s‐PTH), s‐vitamin D, s‐alkaline phosphatase, s‐total cholesterol, s‐glucose, b‐glycosylated hemoglobin A1c. We measured height and weight in all patients at both time points. All patients included in this study received chemotherapy according to applicable local guidelines. Furthermore, we assessed RT dosage due to a known risk of development of hypothyroidism after BC‐related RT.^19^

According to local guidelines, high‐dose peroral corticosteroids are indicated for the prevention of nausea and vomiting concerning chemotherapy and the prevention of infusion reactions during taxane‐related chemotherapy. The patients treated with the combination of paclitaxel and epirubicin + cyclophosphamide received methylprednisolone treatment during the first 4 cycles of paclitaxel treatment (40, 40, 20, and 20 mg, respectively), whereas 50 mg prednisolone concerning 4 cycles of epirubicin + cyclophosphamide treatment was administered. In patients treated with docetaxel and cyclophosphamide in 6 cycles 100 mg of prednisolone before and on the day of chemotherapy was administered, whereas 50 mg of prednisolone 1 day later, and 25 mg of prednisolone 2 and 3 days after chemotherapy was administered.

To assess the possible influence of BC‐related RT on thyroid function we calculated the radiation dosage of the radiation fields to the chest and neck. In our cohort, patients received three types of RT for BC:

1. Locoregional (radiation is given to the ipsilateral breast or chest wall as well as the peri clavicular, and/or parasternal lymph nodes with or without involving axillary lymph nodes)
2. Whole breast (radiation field includes the whole breast)
3. Partial breast (radiation field includes ¼ of the breast)

We have calculated radiation dosage only for patients receiving locoregional RT with involvement of supraclavicular level and thus a risk for the development of hypothyroidism (“high‐risk” patients). Neither whole‐breast‐ nor partial‐breast irradiation involves the thyroid gland. Both mean radiation doses in gray (Gy) and index of equivalent total doses in 2 Gy fractions (EQD2) were calculated. EQD2 index is defined as the dose to be delivered with 2 Gy per fraction to obtain the same biological effect as the treatment under consideration obtains. For conversion, the EQD2 linear‐quadratic model was used (*α*/*β* = 3 Gy).

### 2.3 Statistics

We aimed to detect an s‐TSH change within 0.6 miU/L, as s‐TSH is the most sensitive parameter of thyroid function. For a power of 95%, we needed 61 participants with thyroid measurements before and after chemotherapy. We included 72 patients in our trial based on our power calculation. Categorical data are presented as n (%), continuous as mean (±SD) if normally distributed, and otherwise as median (range). Statistical analysis was performed in RStudio (version 2022.12.0, build 353, “Elsbeth Geranium” Release for Windows). Statistical difference was considered significant when *p* < 0.05. Due to repeated measurements with the same subject, we used the Wilcoxon signed‐rank test, since levels of hormones were not normally distributed. Furthermore, we used a one‐way analysis of variance (ANOVA) to assess the correlation between the difference of s‐TSH change on weight change before‐ and after the course of chemotherapy. At last, we used the Mann–Whitney *U*‐test to assess the difference in thyroid function of patients evaluated <3 months versus >3 months post‐chemotherapy.

## 3 RESULTS

Disease and treatment characteristics are shown in detail in Tables 1 and 2. Almost all EBC patients suffered invasive ductal carcinoma (88%) and malignancy grade II disease (53%) with involvement of 1–3 lymph nodes (47%) (Table 1). The majority had the combination of an estrogen‐receptor‐positive and HER‐2 negative receptor status. Most patients had their tumors removed by lumpectomy (62%) and 85% of the patients received an anthracycline/taxane‐based chemotherapy in combination with cyclophosphamide (Table 2). Anti‐estrogen treatment was indicated in 83% of patients, whereas 40% received trastuzumab treatment (Table 2).

**TABLE 1:** Disease characteristics, cancer type, and staging of the patient group with early breast cancer.

Disease characteristics	*n* = 72
*Histology*	
IDC^a^	63 (88%)
ILC^b^	7 (10%)
Unknown	2 (3%)
*Malignancy grade*	
I	13 (18%)
II	38 (53%)
III	20 (28%)
Unknown	1 (1%)
*Lymph nodes status*	
0	24 (33%)
1–3	34 (47%)
4+	5 (8%)
Unknown	9 (12%)
*Estrogen*‐*receptor status*	
Positive	60 (83%)
Negative	12 (17%)
*HER*‐*2* ^c^ *status*	
Positive	25 (35%)
Negative	46 (64%)
Unknown	1 (1%)

^a^ IDC: invasive ductal carcinoma.

^b^ ILC: invasive luminal (lobular) carcinoma.

^c^ HER‐2: human epidermal growth factor receptor 2.

**TABLE 2:** Cancer treatment characteristics of the patient group with early breast cancer.

Treatment characteristics	*n* = 72
*Surgery type*	
Lumpectomy	45 (62%)
Mastectomy	25 (35%)
Other	2 (3%)
*Type of chemotherapy*	
Cyclophosphamide	61 (85%)
Epirubicin	55 (76%)
Docetaxel	7 (10%)
Paclitaxel	62 (86%)
Other	1 (1%)
*Anti‐estrogen treatment*	
Aromatase inhibitor	58 (80%)
Tamoxifen	2 (3%)
No	12 (17%)
*Trastuzumab treatment*	
Yes	29 (40%)
No	43 (60%)
*Radiation therapy*	
Yes	59 (82%)
No	13 (18%)
*Type of radiation therapy*	
Locoregional	35 (59%)
Whole‐breast	18 (31%)
Partial‐breast	6 (10%)
*Number of radiotherapy fractions*	
15	47 (66%)
25	12 (34%)

Anthropometry and biochemistry results are shown in Table 3. The average duration of chemotherapy was 114 days (range 55–167). The average age at inclusion was 59 years, suggesting an even age distribution (range 50–69). The average weight at inclusion was 76 kg with no significant change after chemotherapy (+255 g, *p* = 0.75). However, weight changes were bidirectional, ranging from −9 to +20 kg (Table 3). Some significant but non‐clinically relevant changes in safety parameters were observed after chemotherapy (Table 3).

**TABLE 3:** Anthropometry, biochemistry, and thyroid parameters of 72 post‐menopausal women with early breast cancer.

Change	Parameter	Before chemotherapy	After chemotherapy	*p*‐value
	Age (years)	59.3 (50–69)	n/a	n/a
→	Weight (kg)	75.9 (42.4–108)	76.2 (41–120)	0.75*
↓	B‐Hemoglobin (mmol/L)	8.4 (± 0.7)	8.0 (± 0.7)	2.19×10^−6^ ***
↓	s‐Leucocytes (10^−9^/L)	5.7 (± 1.9)	5.0 (± 2.1)	1.08×10^−5^ ***
↓	s‐Albumin (g/L)	39.5 (± 3.5)	37.9 (± 2.9)	0.002**
↑	s‐Creatinine (μmol/L)	67.7 (± 9.8)	69.1 (± 11.5)	0.11*
↓	s‐Ionized calcium (mmol/L)	1.3 (± 0.1)	1.2 (± 0.1)	3.0×10^−5^ ***
↓	s‐Alkaline phosphatase (U/L)	18.9 (± 6.0)	17.3 (± 6.3)	0.004**
↑	s‐25‐OH‐D (nmol/L)	62.9 (± 25.9)	73.1 (± 26.0)	6.69×10^−5^ ***
↑	s‐PTH (pmol/L)	5.3 (± 2.2)	6.2 (± 2.5)	0.02**
→	B‐Glucose (mmol/L)	5.7 (± 0.7)	5.7 (± 1.0)	0.44*
→	B‐HbA1c (mmol/mol)	36.5 (± 4.2)	36.0 (± 5.1)	0.39*
→	s‐Total cholesterol (mmol/L)	5.8 (± 1.1)	5.9 (± 0.1)	0.17*
↑	s‐TSH (IU/L)	2.2 ± 1.6	2.6 ± 2.4	0.03**
↓	s‐FT4 (pmol/L) (*N* = 69)	15.4 ± 2.0	14.7 ± 2.0	0.0006***
→	s‐TT4 (nmol/L) (*N* = 68)	93.7 ± 16.0	91.2 ± 16.9	0.06*
↑	s‐TT3 (nmol/L) (*N* = 69)	1.7 ± 0.3	1.8 ± 0.3	0.05*
→	s‐TSH	With RT: 2.42 ± 2.08	With RT: 2.76 ± 3.16	0.64*
		Without RT: 2.15 ± 0.93	Without RT: 2.51 ± 0.34	0.24*

*Note*: Data are given as average (range) or (mean ± SD). The *p*‐value is based on paired analysis (before and after chemotherapy).

Abbreviations: B‐HbA1c, glycated hemoglobin; n/a, not available; s‐25‐OH‐D, 25‐hydroxy‐vitamin D; s‐PTH, parathyroid hormone; s‐TSH, thyroid‐stimulating hormone; s‐FT4, free‐thyroxine; s‐TT4, total thyroxine; s‐TT3, total triiodothyronine; RT, radiotherapy; →, unchanged value; ↓, decreasing value; ↑, increasing value.

* Statistically non‐significant (*p* > 0.05).

** Statistically significant (*p* < 0.05).

*** Statistically highly significant (*p* < 0.001).

**FIGURE 2:**
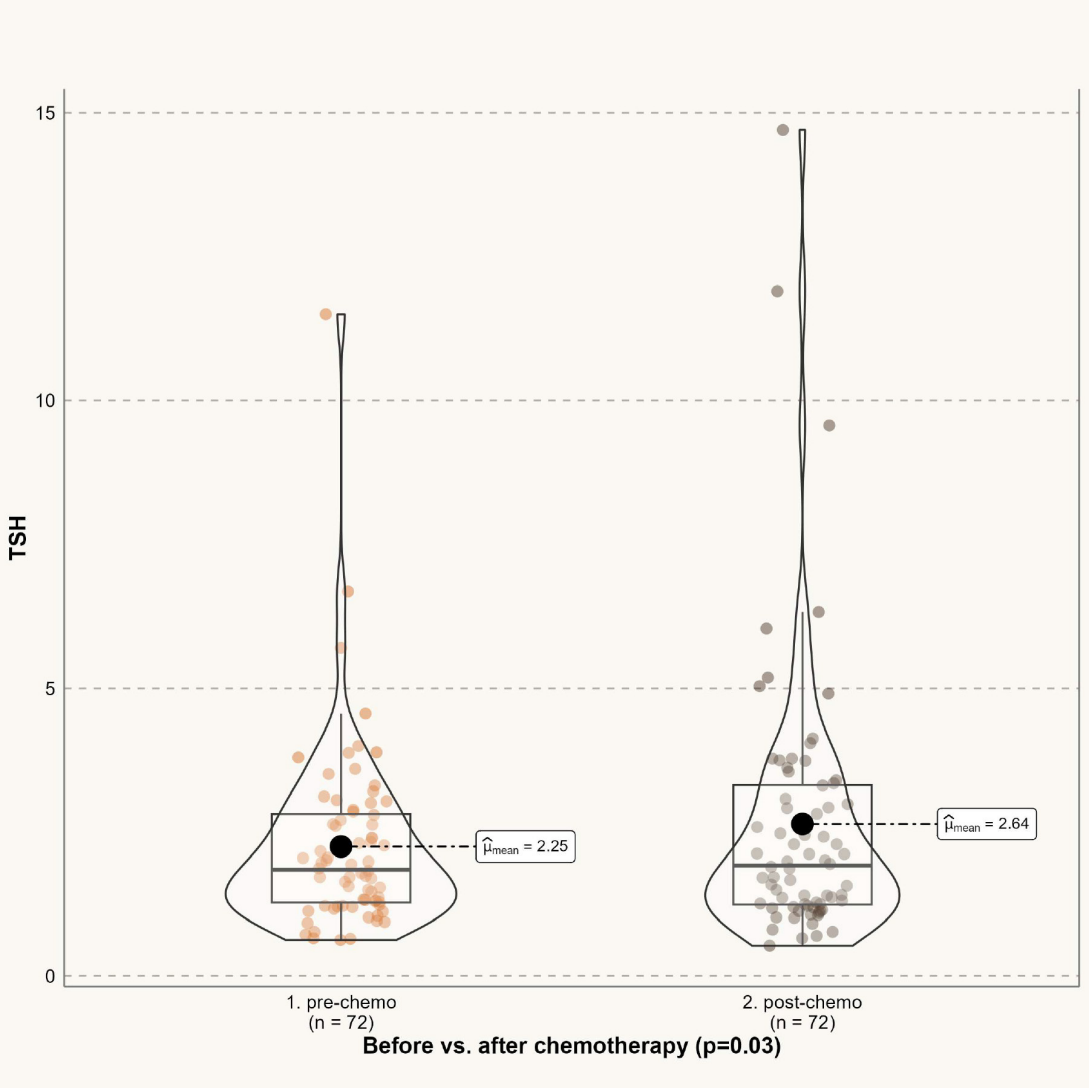
Mean TSH values before and after the course of chemotherapy. TSH, thyroid‐stimulating hormone.

**FIGURE 3:**
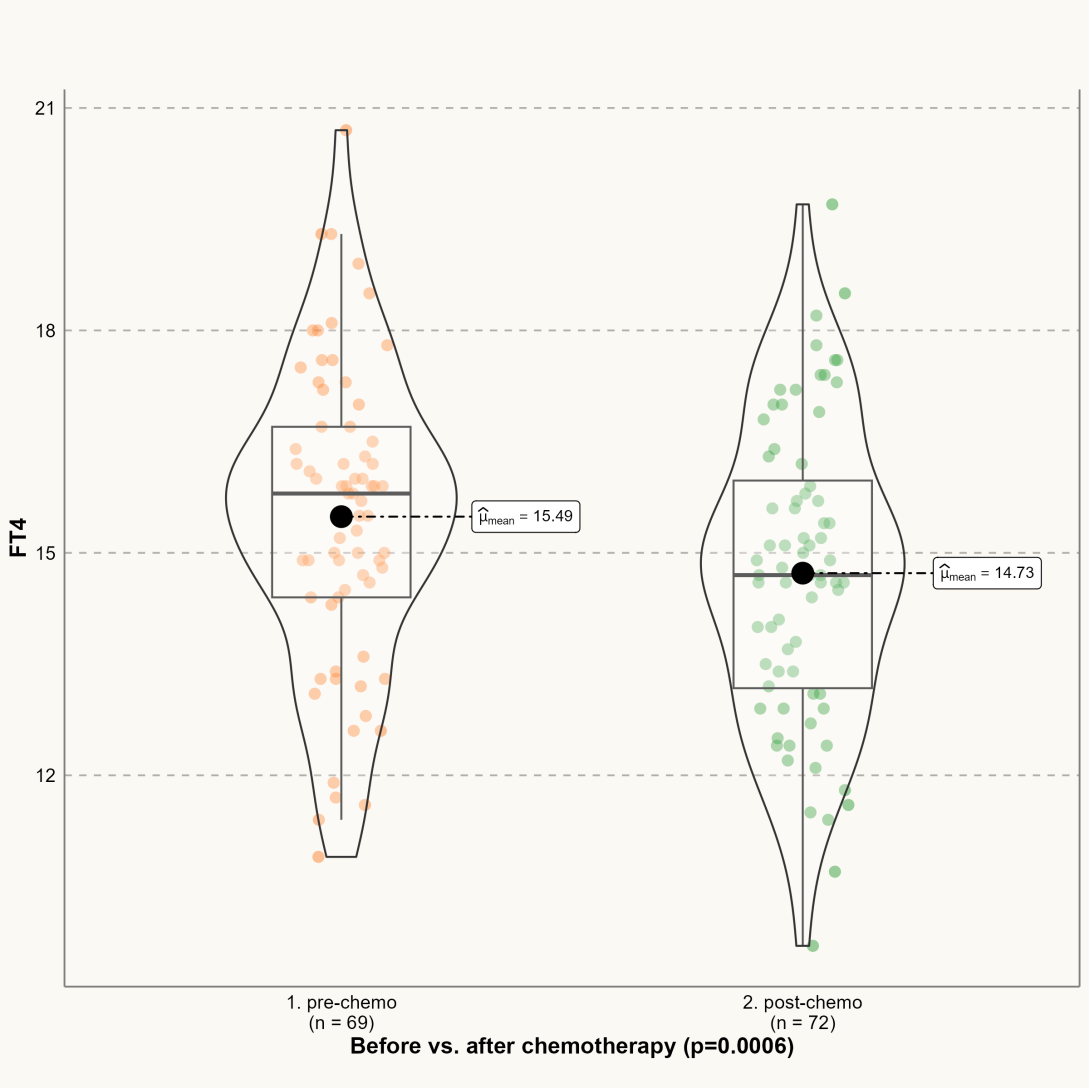
Mean FT4 values before and after the course of chemotherapy. FT4, free‐thyroxine.

Our group evaluation showed a minimal decrease in thyroid function after chemotherapy i.e., s‐TSH increased slightly and significantly (*p* = 0.03), whereas s‐FT4 decreased (*p* = 0.0006) (Table 3). However, the changes were not clinically relevant. Accordingly, s‐TT4 also trended towards a slight decrease (*p* = 0.06) (Table 3). Data on thyroid hormones before and after adjuvant chemotherapy are presented in Figures 2 and 3 (s‐TSH, and s‐FT4, respectively).

We aimed to evaluate most patients within 3 months from termination of chemotherapy. However, some patients did not respond within 3 months to our invitation letter and were assessed later than anticipated, mostly due to the Corona epidemic or personal reasons. As a result, the average time from last chemotherapy to the evaluation of thyroid hormones was 86 days (range 14–190). In total, 44 of 72 patients were evaluated within 3 months after termination of chemotherapy, whereas 28 of 72 patients more than 3 months post‐chemotherapy. We found no difference in s‐TSH values between the patients evaluated <3 months and >3 months of termination of chemotherapy that is, the two groups did not differ at baseline (*p* = 0.44) and after chemotherapy (*p* = 0.92). Furthermore, we did not observe any significant change in TSH from baseline to post‐chemotherapy in any of the two groups (*p* = 0.79, and *p* = 0.25, respectively).

Moreover, at initial evaluation, one patient was diagnosed with overt hypothyroidism (TSH 11.5 × 10^−3^ IU/L) and she initiated treatment with levothyroxine, whereas another patient had subclinical hypothyroidism (TSH 6.68 × 10^−3^ IU/L) and initiated therapy with levothyroxine after adjuvant treatment due to an increasing s‐TSH to 14.7 × 10^−3^ IU/L. After adjuvant treatment, we observed another three patients (4%) with measurements of thyroid function indicating subclinical hypothyroidism (TSH 5.04 × 10^−3^ IU/L, 6.04 × 10^−3^ IU/L, and 6.33 × 10^−3^ IU/L, respectively). A decrease in TT3 was not observed (Table 3). In total, five patients (7%) changed from normal thyroid function to subclinical or overt hypothyroidism after chemotherapy. In these five patients, weight change was observed as follows: −9, −3, +3, +6, and −7.6 kg, respectively, meaning these patients had an average weight loss of −2.12 kg despite diagnosed thyroid dysfunction. None of the patients in our cohort received previous therapy with an immune checkpoint inhibitor or were diagnosed with thyroiditis during RT. Furthermore, few patients were examined with bone scintigraphy due to the impression of bone metastasis, which had not been diagnosed.

In most of the BC patients, thyroid autoantibodies have been evaluated. Positive s‐TgAb was registered in 9 of 66 patients (14%) before chemotherapy, and in 8 of 63 patients (13%) after adjuvant treatment. The same occurrence of positive s‐TPOAb was observed before chemotherapy (9 of 66 patients (14%)), whereas in fewer patients after chemotherapy (4 of 63 patients (6%)). However, the therapy with levothyroxine was not indicated (normal s‐TSH). Only 1 of 58 patients (2%) had slightly elevated s‐TRAb before chemotherapy, and 3 of 67 patients (4%) after chemotherapy. All patients had normal thyroid function at both visits.

RT was administered to 59 of 72 patients (82%) in combination with chemotherapy (Table 2). However, locoregional RT, as a risk factor for hypothyroidism, has been administered in 35 of 59 patients (59%), meaning 41% of RT‐treated patients were non‐risk patients for the development of hypothyroidism. We calculated an average of median values of Gy used in the thyroid region only in the “high‐risk” patients. An average of 10.7 Gy (1.3–23.4 Gy) has been used divided into 25 therapy fractions (34%) or 15 therapy fractions (66%) (Table 2). When EQD2 was calculated, the average of 7.9 Gy (0.8–18.4 Gy) in 35 “high‐risk” patients was administered to the neck region (thyroid). No statistical difference in the TSH change was observed in the subgroup of women treated with locoregional RT compared to the group of women not treated with RT (*p* = 0.64). Furthermore, no significant correlation was observed between the change in s‐TSH versus the change in weight before‐ and after chemotherapy (*R* ^2^ = 0.005 (*p* = 0.52)).

## 4 DISCUSSION

In this study, we investigated whether adjuvant chemotherapy with or without locoregional RT influenced thyroid function in postmenopausal women diagnosed with EBC. A significant but not clinically relevant increase in s‐TSH was observed in combination with a significant decrease in s‐FT4. These data suggest a minimal and not clinically significant effect on thyroid function after chemotherapy and RT. When we compared the subgroup of EBC patients who received locoregional RT to the EBC patients who did not receive RT, we observed no significant difference in s‐TSH.

In 2021, the average self‐reported body weight of 35.927 Danish women aged 50–70 years was 73.5 kg,^25^ whereas the average weight was approximately 76 kg in our study group. We observed no significant weight gain after chemotherapy (average of +255 g (range: −9 to +20 kg)). Four of 72 patients had very low pre‐ and post‐chemo weight, being possible outliers, influencing the overall average weight gain we observed. We applied a sensitivity analysis by excluding four patients with very low weight (body mass index (BMI) <20 kg/m^2^) from the dataset, which did not significantly influence the earlier observed pattern of changes in TSH, FT4 or weight. However, weight gain is thought to be a well‐established long‐term consequence of BC treatments.^11^ Obesity is a risk factor for BC incidence and recurrence, as described in retrospective studies.^26^, ^27^ A recent review confirmed an increased risk of BC in obese patients, probably being driven by the interplay between obesity‐related chronic inflammation, adipokines, insulin resistance, and altered microbiome.^28^ Our results showed no correlation between observed changes in s‐TSH on weight change in our patient group.

Although the results of our study show a modest decrease in thyroid function after chemotherapy, the mechanism behind it, and the clinical significance are limited. A recent meta‐analysis concluded the highest risk of hypothyroidism after radiation in supraclavicular fields.^19^ In one study, an excess risk of hypothyroidism more than 8 years after chemotherapy was registered in patients receiving additional RT.^29^ However, the average time from RT to the development of hypothyroidism is variable—from 8 to 24 months.^30^ Among our 72 included BC patients, 59 received RT, however, only 35 patients (59%) received locoregional RT with involvement of supraclavicular level, thereby being the “high‐risk” patients. In this “high risk” BC patient subgroup we found no statistically significant change in s‐TSH when compared to the group who were not treated with RT. Further hereto, glucocorticoids may affect thyroid function. High‐dose corticosteroids can suppress TSH secretion at the pituitary level,^31^ whereas administration of oral dexamethasone and intravenous betamethasone reduces s‐TT3 and s‐TSH during both administrations, whereas s‐TT4 was reduced after intravenous administration only.^32^ These data are not in line with the observed changes in our EBC patient group, with the observation of statistically significant elevation of s‐TSH after chemotherapy and orally administered prednisolone.

A recent systematic review assessed thyroid function after chemotherapy in 8 studies published until 2019.^18^ Overall, a decrease in s‐TT4, s‐TT3, s‐FT4, and free triiodothyronine (s‐FT3) was registered, whereas alteration of s‐TSH was not clear.^18^ Most studies evaluated s‐TSH before and after the chemotherapy, whereas one publication evaluated thyroid hormones up to 10 months after chemotherapy. Thyroid autoantibodies were not assessed.^18^ Other studies have reported on marginal increase in the risk for hypothyroidism compared to healthy controls on a median of 10.2 years after non‐metastatic BC.^33^ Among these studies De Groot et al. observed a significant increase in s‐TSH and a decrease in s‐FT4 during 6 cycles of chemotherapy,^34^ being in line with our results. In another study including 75 chemotherapy‐treated BC patients average s‐TPOAb concentrations and median s‐TSH were much higher in the chemotherapy‐treated group.^35^ In our study, we observed the combination of decreasing thyroid function after chemotherapy and a prevalence of autoimmune thyroid disease in line with the incidence of autoimmune thyroid disease of 12% in the background population of women in Denmark aged 50–70 years.^36^ With a low prevalence, trastuzumab treatment can also contribute to a risk of autoimmune thyroiditis. European Medicines Agency published data on adverse events in 1.3% of the patients in both subcutaneous and intravenous administration of trastuzumab; however, autoimmune thyroiditis was low‐frequent (0.3% of the patients), and only detected in the intravenous arm.^37^ To date, only a few case reports have been published on trastuzumab‐related autoimmune thyroiditis. Recently, authors published on autoimmune thyroiditis in two patients with EBC receiving trastuzumab subcutaneously.^38^ Since BMI influences the plasma concentrations of trastuzumab, the authors speculated on the relation between the toxicity of trastuzumab and the low‐to‐normal BMI of these two patients (22.4 and 25 kg/m^2^, respectively).^38^ Another two case reports are published on trastuzumab‐related autoimmune thyroiditis after intravenous administration.^39^, ^40^ The average BMI in our EBC patient group was 27.3 kg/m^2^, and the prevalence of thyroid autoantibodies was in line with the background population in Denmark, thus suggesting that the observed thyroid hormone changes in our patient group were not due to autoimmune thyroiditis related to intravenously administered trastuzumab. Furthermore, individual patients from our cohort were examined by bone scintigraphy during chemotherapy. We do not suggest iodine‐induced alterations in thyroid function due to the known risk of subclinical hyperthyroidism, which was not observed in our cohort. At last, we did not include patients with triple‐negative BC who tend to have more aggressive BC types with poorer prognoses. In these patients, the combination of chemotherapy and treatment with immune checkpoint inhibitors (atezolizumab, pembrolizumab, nivolumab, avelumab, atezolizumab, durvalumab) is often indicated,^41^ which could be a risk factor for autoimmune thyroiditis or hypothyroidism.^42^

Studies with different set‐ups and follow‐ups of the thyroid hormones after chemotherapy suggested contrasting results: incidence of non‐thyroidal illness,^43^ declining FT4 and FT3 without significant change in TSH,^44^ increasing thyroxin‐binding globulin (TBG).^45^ The participating post‐menopausal EBC patients in our study in most cases received cyclophosphamide, paclitaxel, and epirubicin therapy. Reinhardt W. et al. described a significant increase in s‐TT4 and s‐FT4 with a concomitant decrease in s‐TSH 4 days after chemotherapy with cyclophosphamide, thus with another indication than EBC.^46^ These authors suggested thyroid changes are probably related to changes in TBG.^46^ A risk of transient and mild subclinical hypothyroidism in patients treated with tamoxifen has been shown (reviewed in Marina et al.^47^), whereas no changes in thyroid function were found after treatment with letrozole.^47^ Unfortunately, we do not have a measurement of TBG, which may be changed by anti‐estrogen treatment resulting in a reduction of TT4, with no effect on TSH value. Only two patients in our cohort were treated with tamoxifen. We are not aware of any studies evaluating paclitaxel and epirubicin on thyroid function.

Our study group conducted an analysis of thyroid hormones using immunohistochemistry, which was the most affordable assay and readily available at a low cost in our department. However, this assay can be prone to different interferences such as biotin, thyroid autoantibodies, or conditions with lower‐than‐normal TBG, which can lead to false reduced or increased test results for some patients.^48^ Liquid chromatography–tandem mass spectrometry (LC–MS/MS) is a more sensitive method of assessing free‐hormone concentrations. It has been documented that free thyroid hormones measured by LC–MS/MS correlate better with log‐transformed TSH than those measured by immunohistochemistry, particularly in certain patient groups.^49^ The analysis of FT3 was not performed due to the unavailability of the assay for FT3 measurements, which has been abandoned due to imprecise estimation of T3.

Studies to date have observed a variety of thyroid dysfunctions due to chemotherapy and our study supports a minor but not clinically relevant decrease in thyroid function shortly after chemotherapy treatment. We intend to prospectively follow these patients for 5 years, performing a yearly analysis of thyroid hormones and TBG to assess the long‐term changes in thyroid function.

Our study has strengths and limitations. One limitation is that we did not include an age‐matched control group. Another limitation is the lack of data on TBG since the observed changes in thyroid function could partially be due to changes in TBG. Furthermore, we evaluated the treatment‐induced effect on thyroid function on average 86 days after the patients had completed chemotherapy, which might affect the thyroid function towards normalization. The exclusion of patients with triple‐negative BC treated with chemoimmunotherapy, and the lack of FT3 measurement are additional limitations; however, we aimed to primarily calculate the “true” and not estimated FT3, thus equilibrium dialysis LC–MS/MS was not accessible at our department. At last, a robust conclusion on the influence of RT on thyroid function is difficult to generate as a longer follow‐up of thyroid hormones after completed RT is needed.

However, our study has also some strengths, even though different chemotherapy treatments were used, as we included only patients in the early stage of the disease, thus reducing the risk of low T3 syndrome as a cause of thyroid dysfunction. The few observations of changes in thyroid function did not differ from the risk of hypothyroidism among the background population of women aged 50–70. Due to the prospective nature of our study, we will be able to make a longer follow‐up of thyroid function in 5 years after chemotherapy and RT.

## 5 CONCLUSION

In conclusion, our study shows a statistically significant although not clinically relevant decrease in thyroid function among post‐menopausal women with EBC shortly after chemotherapy. It is unknown whether the thyroid dysfunction after chemotherapy and radiation is transient or will become clinically significant over time. Longer prospective studies on thyroid hormone changes and measurements of TBG are needed to clarify the significance of these observations.

## AUTHOR CONTRIBUTIONS

**Djordje Marina:** Conceptualization (lead); data curation (lead); formal analysis (lead); investigation (equal); methodology (equal); project administration (equal); validation (lead); visualization (equal); writing – original draft (lead); writing – review and editing (lead). **Kristian Buch‐Larsen:** Conceptualization (equal); investigation (equal); methodology (equal); writing – review and editing (equal). **Linn Gillberg:** Methodology (supporting); validation (supporting); writing – review and editing (supporting). **Mads Albrecht Andersen:** Data curation (supporting); formal analysis (supporting); writing – review and editing (supporting). **Michael Andersson:** Methodology (supporting); supervision (supporting); writing – original draft (supporting); writing – review and editing (supporting). **Åse Krogh Rasmussen:** Methodology (equal); project administration (equal); supervision (equal); validation (equal); writing – original draft (equal); writing – review and editing (equal). **Peter Schwarz:** Conceptualization (equal); data curation (equal); formal analysis (equal); investigation (equal); methodology (equal); project administration (lead); supervision (lead); writing – original draft (equal); writing – review and editing (equal).

## FUNDING INFORMATION

This study was financially supported by the Svend Andersen Fond, Aase and Ejnar Danielsens Fond, Kirsten and Freddy Johansen Fond, and Martha and Hans Peter Johnsens Fond. Neither had a role in writing the manuscript or the decision to submit it for publication.

## CONFLICT OF INTEREST STATEMENT

The authors declare that there is no conflict of interest that could be perceived as prejudicing the impartiality of the research reported.

## ETHIC STATEMENT

The study complied with the Declaration of Helsinki, and was approved by the Ethical Committee, Greater Region of Copenhagen (H‐18016600).

## CONSENT

All participants provided signed informed consent before study participation.

## CLINICAL TRIAL REGISTRATION

This study is registered at clinicaltrials.gov (NCT03784651).

## Supporting information


Table S1.

## Data Availability

Data sharing of published basic data is available upon request.
