# Correction to “Chemotherapy for Post‐Menopausal Women With Early Breast Cancer Seems Not to Result in Clinically Significant Changes in Thyroid Function”

**DOI:** 10.1002/cam4.70766

**Published:** 2025-03-15

Marina D, Buch‐Larsen K, Gillberg L, et al. Chemotherapy for post‐menopausal women with early breast cancer seems not to result in clinically significant changes in thyroid function. *Cancer Med*. 2024; 13:e70015. doi:10.1002/cam4.70015

The authors have unfortunately identified an error in the **Methods section (page 3 of 12)**, where we incorrectly described the assay as **“immunohistochemistry” instead of the correct term “immunoassay.”** The accurate sentence should read “Thyroid parameters (s‐TSH, s‐TT4, s‐FT4, s‐TT3) were analyzed by **immunoassay**; s‐TgAb and s‐TPOAb were analyzed by immunofluorometry, whereas s‐TRAb was analyzed by electrochemiluminescence immunoassay.”

Additionally, this error appears two times in the **Discussion section (page 9 of 12)**, where **“immunohistochemistry” should also be replaced with “immunoassay.”** The accurate sentences should read:

“Our study group conducted an analysis of thyroid hormones using **immunoassay**, which was the most affordable assay and readily available at a low cost in our department.”

“It has been documented that free thyroid hormones measured by LC–MS/MS correlate better with log‐transformed TSH than those measured by **immunoassay**, particularly in certain patient groups.”

Furthermore, in **Supplementary Table S1 (page 3 out of 12)**, the assay type for the measurements of TSH, TT4, FT4, and TT3 should be **corrected again from “immunohistochemistry” to “immunoassay.”** The corrected table can be found in the online Supporting Information.

We sincerely apologize for this error.

