# Supplementary material for: Chemotherapy for post‐menopausal women with early breast cancer seems not to result in clinically significant changes in thyroid function
Source: Cancer Med. 2024 Aug 6;13(15):e70015. doi: 10.1002/cam4.70015 (PMC11303825; doi:10.1002/cam4.70015)
Supplement: Supplementary file 1 — Table S1. [file CAM4-13-e70015-s001.docx]

**Supplementary Table 1. Assays used for the serum hormone measurements**

| *Hormone measurement* | *Assay type and equipment* | *Reference values* | *Detection*  *limit* | *Intraassay imprecision* | *Total assay imprecision* |
| --- | --- | --- | --- | --- | --- |
| TSH† | Immunohistochemistry  (Cobas) | 0.40 – 4.80 x 10^-3^  IU/L | 0.01 – 1000 x 10^-3^ IU/L | 4% | 14% |
| TT4‡ | Immunohistochemistry  (Cobas) | 15 – 320  nmol/L | 70.0 – 140.0  nmol/L | 7% | 16% |
| FT4§ | Immunohistochemistry  (Cobas) | 12 – 22  pmol/L | 1.3 – 100  pmol/L | 6% | 14% |
| TT3¶ | Immunohistochemistry  (Cobas) | 1.4 – 2.8  nmol/L | 0.4 – 10.0  nmol/L | 6% | 14% |
| TgAb^ | Immunofluorometry  (Cryptor Compact Plus) | < 33  IU/L | 33 – 20.000  IU/L | 5% | 2 x 7.1% |
| TPOAb¤ | Immunofluorometry  (Cryptor Compact Plus) | < 60  IU/L | 50 – 20.000  IU/L | 8% | 2 x 10.6% |
| TRAb⁋ | Electrochemiluminescence  immunoassay (ECLIA)  (Cobas) | < 1.0  IU/L | 0.8 – 40  IU/L | 10% | 20% |

†TSH: thyroid-stimulating hormone

‡TT4: total thyroxine

§FT4: free-thyroxine

¶TT3: total triiodothyronine

^TgAb: thyroid-globulin antibody

¤TPOAb: thyroid-peroxidase antibody

⁋TRAb: thyroid-stimulating hormone-receptor antibody
